# The two peptide lantibiotic lacticin 3147 acts synergistically with polymyxin to inhibit Gram negative bacteria

**DOI:** 10.1186/1471-2180-13-212

**Published:** 2013-09-26

**Authors:** Lorraine A Draper, Paul D Cotter, Colin Hill, R Paul Ross

**Affiliations:** 1 Department of Microbiology, University College Cork, Cork, Ireland; 2 Alimentary Pharmabiotic Centre, University College Cork, Cork, Ireland; 3 Teagasc Food Research Centre, Moorepark, Fermoy, County Cork, Ireland

**Keywords:** Antimicrobial, Synergy, Lantibiotic, Bacteriocin, Lacticin 3147, Polymyxin

## Abstract

**Background:**

The emergence of bacterial drug resistance encourages the re-evaluation of the potential of existing antimicrobials. Lantibiotics are post-translationally modified, ribosomally synthesised antimicrobial peptides with a broad spectrum antimicrobial activity. Here, we focussed on expanding the potential of lacticin 3147, one of the most studied lantibiotics and one which possesses potent activity against a wide range of Gram positive species including many nosocomial pathogens. More specifically, our aim was to investigate if lacticin 3147 activity could be enhanced when combined with a range of different clinical antibiotics.

**Results:**

Initial screening revealed that polymyxin B and polymyxin E (colistin) exhibited synergistic activity with lacticin 3147. Checkerboard assays were performed against a number of strains, including both Gram positive and Gram negative species. The resultant fractional inhibitory concentration (FIC) index values established that, while partial synergy was detected against Gram positive targets, synergy was obvious against Gram negative species, including *Cronobacter* and *E. coli*.

**Conclusions:**

Combining lacticin 3147 with low levels of a polymyxin could provide a means of broadening target specificity of the lantibiotic, while also reducing polymyxin use due to the lower concentrations required as a result of synergy.

## Background

The challenge presented by the emerging problem of antibiotic resistance is a significant one. One approach has been to identify new bactericidal agents while another has involved a re-examination of the potential of previously identified antimicrobials. With this latter route in mind, there has been a particular focus on assessing and enhancing the benefits of applying lantibiotics in clinical settings [1,2]. Lantibiotics are ribosomally synthesised antimicrobial peptides that are subjected to post-translational modification, resulting in the presence of unusual amino acids including intramolecular lanthionine and β-methyl lanthionine bridges. These bridges are formed through a two-step process that is initiated by the dehydration of serine and threonine residues to dehydroalanine (dha) and dehydrobutyrine (dhb), respectively. The subsequent reaction of these modified amino acids with intrapeptide cysteines results in the formation of lanthionine (Ala-S-Ala; in the case of dha) or β-methyl-lanthionine (Abu-S-Ala; in the case of dhb) bridges (for reviews see [3-5]). Lacticin 3147 is a two peptide lantibiotic which exhibits broad spectrum activity against Gram positive targets. The two lacticin 3147 peptides, Ltnα and Ltnβ, work synergistically in a 1:1 ratio [5,6]. Ltnα first binds to the precursor of peptidoglycan production, lipid II, with Ltnβ subsequently interacting with this complex. The net effect is the inhibition of peptidoglycan synthesis and the formation of a membrane depolarising pore [7].

Some lantibiotics are active at single nanomolar levels against particular targets and several lantibiotics inhibit drug–resistant Gram positive pathogens, including methicillin-resistant *Staphylococcus aureus* (MRSA) and vancomycin-resistant enterococci (VRE) [1,2]. Lantibiotics are highly stable, resistance is rare and activity can be enhanced through genetic alteration and, thus, they are considered to be viable alternatives to traditional antibiotics [1]. Lacticin 3147 inhibits many Gram positive pathogens including *Listeria monocytogenes*, *Staphylococcus aureus* and *Clostridium difficile* as well as a variety of streptococci, enterococci and mycobacteria [8-10]. However, to date, the inhibition of Gram negative species by lacticin 3147 has not been reported. This is most often attributed to the presence of the outer membrane, which prevents access of the lantibiotic to the cytoplasmic membrane.

There are many potential benefits associated with identifying antibiotics that function synergistically with lacticin 3147. While antibiotic resistance has become a major obstacle, significant resistance to lacticin 3147 has yet to be reported and thus the use of antibiotic-lacticin 3147 combinations may prevent/overcome the emergence of resistance. Furthermore, certain antibiotic-lacticin 3147 combinations may allow for a broader range of species to be targeted. Here we assess the impact of combining lacticin 3147 with a variety of clinical antibiotics and establish that lacticin 3147 exhibits synergistic activity in combination with either polymyxin B or polymyxin E.

## Results

### Sensitivity of bacteria to lacticin 3147 and antibiotics in combination

**Figure 1:**
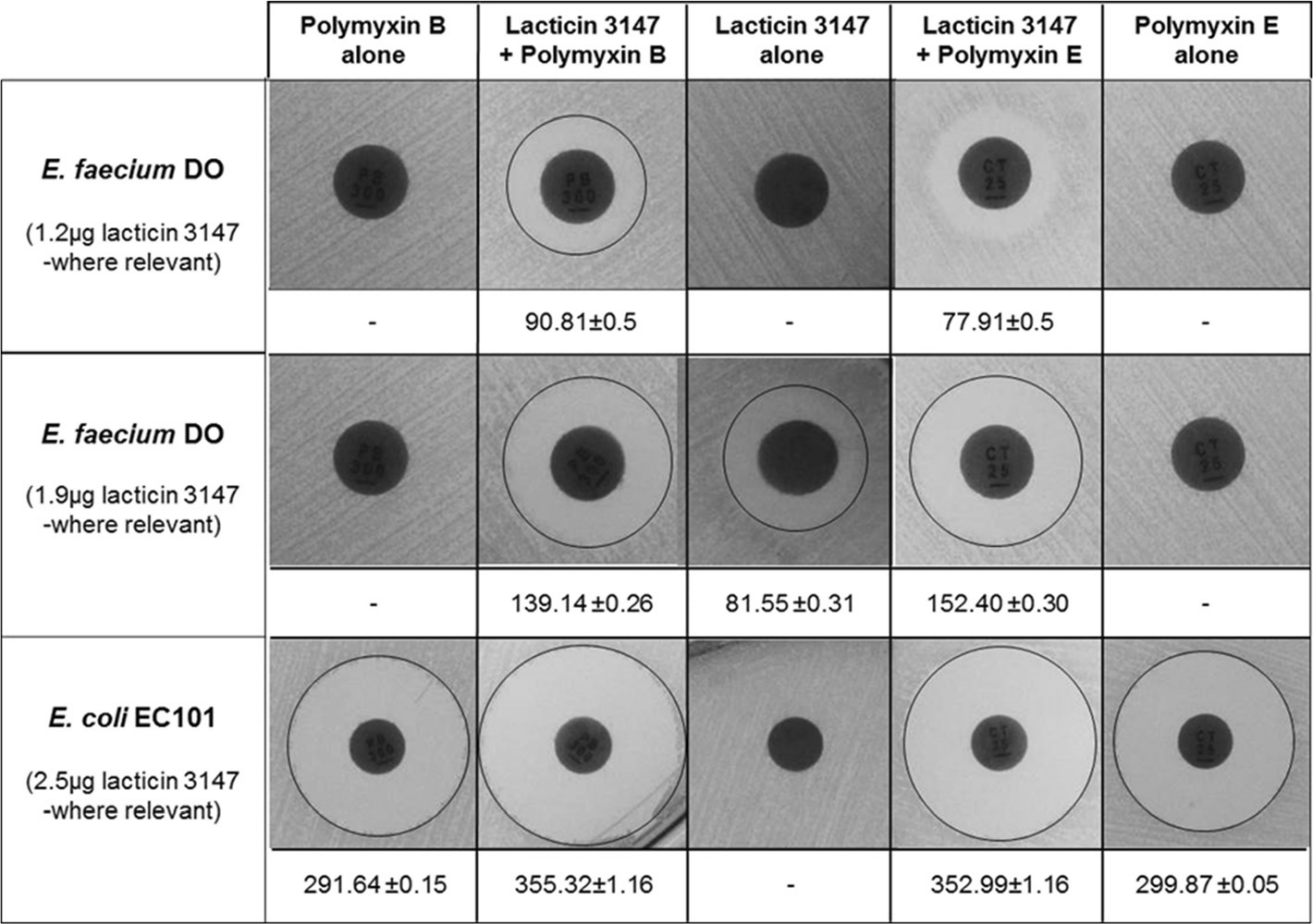
**Antibiotic disc-based assessment of lacticin 3147 and polymyxin B/E sensitivity and synergy.** Antibiotic discs infused with polymyxin B and polymyxin E were placed on agar plates swabbed with *E. faecium* DO and *E. coli* EC101. Lacticin 3147 (1.2, 1.9 or 2.5 μg) was added to additional discs containing the respective polymyxins and to blank, non-polymyxin containing, controls. Results are the outcome of duplicate experiments and are expressed as total area of inhibitory zone expressed in mm^2^.

To determine whether lacticin 3147 could work synergistically with a variety of clinically utilised antibiotics, we used antibiotic disc assays to assess the potency of individual antibiotics (cefotaxime, novobiocin, cefoperazone, teicoplanin, ceftazidime, cefaclor, cephradine, cefaclor (30 μg), bacitracin, imipenem, fusidic acid (10 μg), penicillin G (5 μg), oxacillin (1 μg), colistin sulphate (polymyxin E) (25 μg) and polymyxin B (300 U)), in the presence and absence of lacticin 3147. It was evident that lacticin 3147 had the ability to enhance the activity of a number of the antibiotics tested (data not shown) but the benefits of combining lacticin 3147 with polymyxin B or polymyxin E were particularly obvious (Figure 1). In the case of the representative Gram positive and negative strains, *E. faecium* DO and *E. coli* EC101, the diameters of the zones of inhibition were increased by over 180% and by over 121%, respectively. Indeed, in the case of *E. faecium* DO, combining sub-inhibitory concentrations of the individual antimicrobials resulted in the formation of a zone of clearing (Figure 1). Based on these preliminary experiments it was apparent that the benefits of combining lacticin 3147 with either polymyxin B or E merited further examination. We used broth based microtitre plate assays to determine minimum inhibitory concentrations (MICs) and combined FICs against a range of Gram negative and representative Gram positive strains (Table 1). It was apparent that a combination of lacticin 3147 and polymyxin B or E had an indifferent effect (FIC = 1.25 and 1.125 respectively) against *Salmonella* Typhimurium UK1 and an antagonistic effect (FIC > 4) was observed in the case of the LT2 strain. However, combining these antimicrobials against other targets gave more positive results. Indeed, a high level of synergy was observed against *Cronobacter sakazakii* strain 6440, with an FIC index corresponding to 0.250 for a lacticin 3147 and polymyxin B combination and 0.062 for a lacticin 3147 and polymyxin E combination. FIC values here were determined on the basis of the reduction in MIC values for the polymyxins alone as an MIC value for lacticin 3147 could not be determined as it is not active against *C. sakazakii*, even at the highest level tested (924 μg/ml). However, it can be established that the FIC is <0.312 for lacticin 3147 in combination with polymyxin B and <0.125 when combined with polymyxin E.

**Table 1:** MIC data for lacticin 3147, polymyxin B and polymyxin E alone and in combination

**Organism**	**MIC (μg/ml)**						
	**Lacticin 3147**	**Polymyxin B**	**Polymyxin E**	**Lacticin 3147/**	**FIC**	**Lacticin 3147/**	**FIC**
				**Polymyxin B**		**Polymyxin E**	
*Salmonella* Typhimurium UK1	924	0.0586	0.0586	924/0.015	1.25^d^	924/0.0073	1.125^d^
*Salmonella* Typhimurium LT2	231	0.3125	0.4688	No MIC	>4^e^	No MIC	>4^e^
*Cronobacter sakazakii DPC* 6440	>924	0.3125	0.3125	57.75/0.0781	0.250 (<0.312)*^a^	57.75/0.0195	0.062 (<0.125)*^a^
*E. coli* 0157:H-	231	0.0586	0.0781	28.875/0.0073	0.250^a^	28.875/0.0049	0.188^a^
*E. coli* DH5α	462	0.0781	0.0781	28.875/.0098	0.188^a^	28.875/0.0098	0.188^a^
*E. coli* EC101	462	0.0781	0.0781	14.4375/.0391	0.5^a^	28.875/0.0098	0.188^a^
*E. faecium DO*	0.9625	>375	>375	0.9625/23.4375	1^c^	0.9652/23.4375	1^c^
*B. cereus 8079*	3.85	187.5	375	1.925/23.4375	0.62^b^	3.85/375	2^d^
*S .aureus 5247*	15.4	187.5	>375	7.7/46.875	0.75^b^	15.4/23.4375	1^c^

FIC figures have been calculated as a result of triplicate experiments and indicate ^a^synergy, ^b^for partial synergy, ^c^additive effects, ^d^indifference, and ^e^antagonism between the combined antimicrobials. *FIC index which includes the reduction in lacticin 3147 MIC from the highest level tested to that which achieves an MIC in the presence of polymyxin.

Corresponding studies with three *E. coli* strains again revealed synergism between lacticin 3147 and the polymyxins. An FIC index value of 0.248 was obtained when lacticin 3147 and polymyxin B were combined against 0157:H- while the corresponding lacticin 3147 and polymyxin E FIC value was 0.188. When lacticin 3147 and polymyxin B were combined against *E. coli* DH5α and EC101, FIC indices of 0.188 and 0.5 were obtained, respectively. In addition, an FIC index of 0.188 was determined when lacticin 3147 and polymyxin E were combined for these two target strains.

A number of additional assays were carried out in order to determine if the benefits of combining lacticin 3147 and the polymyxins in broth extended to Gram positive targets. For this purpose *Bacillus cereus* 8079, *Enterococcus faecium* DO and *Staphylococcus aureus* 5247 were selected as representative indicator strains. It was established that, while some partial synergy between lacticin 3147 and polymyxin B was observed with respect to *B. cereus* 8079 and *S. aureus* 5247 (FIC = 0.62 and 0.75, respectively), the other combinations resulted in an additive or indifferent outcome.

Given that the most notable outcome from the study was the synergistic activity of lacticin 3147 and the polymyxins against some Gram negative targets, further investigations were carried out to determine how the respective components of lacticin 3147, i.e. Ltnα and Ltnβ, perform individually in the presence of polymyxin B/E. Selecting the sensitive strain *E. coli* 0157:H- as a target, we were able to evaluate the contribution of the individual α and β peptides to this phenomenon (Table 2). Taking into consideration the molecular weights and 1:1 ratio at which α and β are combined, we can derive the relative amount (μg/ml) of each individual peptide present when lacticin 3147 (Ltnα and Ltnβ combined in a 1:1 ratio) is synergistic with polymyxin B/E. With this information we can compare the action of α and β alone to the same amount of each peptide present in whole lacticin 3147 in each case of synergy. Although various degrees of synergy exist due to the different combinations and concentrations assessed, only those that yielded the greatest synergy with respect to lacticin 3147 are listed in Table 1. Obtaining such a high degree of synergy was not possible with the single peptides, Ltnα and Ltnβ. For this reason additional synergy values/FIC data for lacticin 3147 in combination with polymyxin B and E has been included in Table 2. This provides a means by which the contribution of the individual lacticin 3147 components can be derived by focusing on a fixed level of polymyxin B/E in each case of synergy. Hence, it is apparent that, when combined with a set concentration of polymyxin B and E, 6 times more Ltnα alone is required to achieve the level of synergy obtained when both Ltnα and Ltnβ are present. In contrast, only 4.7 times Ltnβ alone is required to achieve a corresponding level of activity in the absence of Ltnα. Interestingly the reverse is seen when you consider the individual action of Ltnα and Ltnβ alone, in the absence of polymyxin. In this situation only 1.5 times the amount of Ltnα is required, while 4.7 times Ltnβ is needed to achieve an MIC relative to their contribution when both lacticin 3147 peptides are present.

**Table 2:** MIC data for lacticin 3147, and its individual peptides Ltnα and Ltnβ, polymyxin B and polymyxin E alone and in combination

***E.coli *0157:H-**						
**MIC (μg/ml)**						
Lacticin 3147	Polymyxin B	Polymyxin E	Lacticin 3147/	**FIC**	Lacticin 3147/	**FIC**
			Polymyxin B		Polymyxin E	
231 (37.5 μM)	0.0586	0.0781	28.875/0.0073	0.250^a^	28.875/ 0.0049	0.188^a^
(α :124.74, Β: 106.26)			28.875 / 0.0147*	0.376*^a^	14.4375 / 0.0195*	0.312*^a^
Ltnα	Polymyxin B	Polymyxin E	Ltnα/	FIC	Ltnα/	FIC
			Polymyxin B		Polymyxin E	
187.11 (56.25 μM)	0.0586	0.0781	93.555 / 0.0073	0.625^b^	46.7775/ 0.0195	0.500^a^
(**1.5** X Ltnα)			(**6.0** X Ltnα in combin.)		(**6.0** X Ltnα in combin.)	
Ltnβ	Polymyxin B	Polymyxin E	Ltnβ/	FIC	Ltnβ/	FIC
			Polymyxin B		Polymyxin E	
495.88 (175 μM)	0.0586	0.0781	61.9850 / 0.0147	0.376^a^	30.9925 / 0.0195	0.313^a^
(**4.7** X Ltnβ)			(**4.7** X Ltnβ in combin.)		(**4.7** X Ltnβ in combin.)	

FIC figures have been calculated as a result of triplicate experiments and indicate ^a^synergy and ^b^partial synergy effects.*Alternative MIC and FIC data that allow for fixed levels of polymyxin across antimicrobial combinations, thus allowing for the calculation of the involvement of Ltnα and Ltnβ in synergy with polymyxin.

## Discussion

We undertook a series of investigations to determine whether lacticin 3147 acts synergistically with a range of clinically important antibiotics. Antibiotics encompassing many families and modes of action were chosen, including cephalosporins, polypeptides, glycopeptides, carbenems, and quinolones. Following this initial screen, it became clear that lacticin 3147 and the polymyxins acted synergistically.

Polymyxins are a group of polypeptide antibiotics that exclusively target Gram negative microorganisms. The five distinct members of this group, polymyxin A-E, were discovered in 1947 and are produced non-ribosomally by different *Bacillus polymyxa* species [11]. Polymyxin B and polymyxin E (also referred to as colistin), have been used in clinical practice for decades in otic and ophthalmic solutions [12,13]. Polymyxins are decapeptide antibiotics which consist of a heptapeptide ring, with polymyxin E differing from polymyxin B only by the presence of D-Leu *in lieu* of a D-Phe. This ring is linked to a tripeptide side-chain which carries an aliphatic chain attached via an amide bond to the amino terminus [14]. The polymyxins carry five positive charges due to the presence of L-α-γ-diaminobutyric acids [11] and it has been established that the amphiphilic nature of this molecule gives it the ability to interact, bind and traverse the Gram negative outer membrane. The target molecule is lipopolysaccharide (LPS) [15], and specifically the lipid A component [16,17]. The polymyxins dissociate protective divalent cations from their association with anionic LPS. This displacement permeabilises the Gram negative outer membrane to allow the polymyxins, or other cationic peptides, to form pores [18]. It should be noted, however, that the use of polymyxins in clinical settings has been restricted to use only where drug resistant pathogens have been encountered. This is due to the toxicity, primarily nephro- and neuro-toxicity, associated with its use [19], although this toxicity has been suggested to be dose dependent [20]. Nonetheless, the polymyxins are, in many cases, the only antibiotics capable of overcoming specific drug resistant pathogens such as *Pseudomonas aeruginosa* and *Acinetobacter baumannii* in cystic fibrosis patients (for reviews see [21-23]). For this reason the polymyxins cannot be ignored, but strategies that could reduce the dose needed for these antibiotics to be effective are highly desirable.

A number of studies have investigated the consequences of combining various antibiotics with polymyxins. Antimicrobial agents such as miconazole [24], rifampicin [25,26] meropenem, ampicillin-sulbactam, ciprofloxacin, piperacillin-clavulanic acid, imipenem, amikacin, and gentamicin [27] ciprofloxacin [28] trimethoprim, trimethoprim-sulfamethoxazole, and vancomycin [29], to name but a few, have been the focus of studies to assess if they can work synergistically with polymyxins (also see Yahav *et. al*., for a review of compounds synergistic with polymyxin E [30]). To date the only lantibiotic to have been investigated in this way is nisin, which displays synergy with polymyxin B and polymyxin E against *Listeria* and *E. coli*[31,32]. Nisin has also been shown to function synergistically when combined with polymyxin E (and clarithromycin) against *Pseudomonas aeruginosa*[33]. Combination studies have also recently revealed that lacticin 3147 and the lactoperoxidase system (LPOS) successfully inhibited growth of *Cronobacter* spp. in rehydrated infant formula [34]. Lacticin 3147, like nisin, is a food grade bactericidal agent obtained from the GRAS organism *Lactococcus lactis.* Notably, however, it differs from nisin with respect to its target specificity and its greater potency against a number of species [10]. Also the mechanism of action contrasts from the single nisin peptide, in that it requires the interaction of two peptides, Ltnα and Ltnβ, for optimal bactericidal activity.

Here, we report the first study to investigate whether synergy can occur between polymyxin(s) and a two-component lantibiotic. Not only do we reveal that synergy is apparent against a range of strains tested, we also investigated the individual contributions of Ltnα and Ltnβ. We established that, when combined with polymyxin B/E, the levels of lacticin 3147 required to inhibit Gram negative species are equivalent or lower than the levels of lacticin 3147 alone against many Gram positive targets. Thus, in the presence of 0.3125 μg/ml polymyxin B/E, the concentration of lacticin 3147 required to inhibit *Cronobacter* spp. is less than the lacticin 3147 MIC for *Mycobacterium avium* subsp. *paratuberculosis* (MAP) ATCC 19698 or *Mycobacterium kansasii* CIT11/06 [8]. Similarly the MIC of lacticin 3147 (alone) against many *S. aureus* (which includes many of the nosocomial pathogens: methicillin-resistant *S. aureus* (MRSA), *S. aureus* with intermediate resistance to vancomycin (VISA), *S. aureus* with heterogenous vancomycin intermediate resistance (hVISA)) [10,35], is greater than that required to inhibit *E. coli* species when in the presence of a polymyxin. It is also important to note that synergy with lacticin 3147 may provide a means of reducing the dose of polymyxins required to inhibit specific targets, thereby addressing polymyxin-associated toxicity issues. For example, 8-fold and 16-fold lower levels of the polymyxins are required to inhibit *E. coli* and *Cronobacter* when in the presence of lacticin 3147. Furthermore a recent study by Naghmouchi *et al*., has shown that in addition to its role in providing synergy with polymyxin E, the lantibiotic nisin appears, at certain concentrations, to eliminate its toxicity, as seen in Vero cell lines [36]. Having established the role lacticin 3147 has in polymyxin synergy, further investigations are warranted in order to ascertain if such toxicity preventing attributes are common amongst lantibiotics.

As with previous studies [37], the solo activities of polymyxin B and polymyxin E against the strains tested here are very similar. With respect to the dual action of lacticin 3147 and polymyxins, it appears that the lacticin 3147-polymyxin B combination has the greater potency against Gram positive targets but that the lacticin 3147-polymyxin E combination has a greater effect against Gram negative strains. Thus, the single amino acid difference between the two polymyxin peptides appears to have an impact on its bactericidal action and target specificity when combined with lacticin 3147. It was also notable that the lacticin 3147 sensitivity of Gram positive microorganisms such as *Enterococcus faecium* DO, which is already highly sensitive to lacticin 3147, is not enhanced by the presence of the polymyxins. However, in the case of the strains that are relatively more lacticin 3147 resistant, the benefits of adding polymyxin B (especially with respect to Gram positive strains) and polymyxin E (especially for Gram negative strains) is most apparent. It is interesting to note that this phenomenon does not correlate with results obtained during the initial agar based disc assay screen, where the opposite pattern was observed. However, it is acknowledged that the agar-based screen is a much cruder assay, and in that instance polymyxin concentrations were fixed and only lacticin 3147 concentrations were altered. Moreover, no FIC data can be derived and so increased zone sizes may not represent the optimal combination of the antimicrobials as obtained through checkerboard assays. The mechanism by which this synergy occurs with respect to Gram negative targets is presumably based on the action of polymyxin permeabilising the outer membrane to allow lacticin 3147 to gain access to the cytoplasmic membrane and its lipid II target [38]. However, a phenomenon concerning the synergy between polymyxin B/E and the singular peptides Ltnα and Ltnβ is also unveiled during this study. Considering the action of the singular peptides in the absence of polymyxin, a greater quantity of Ltnβ alone, than Ltnα alone, is required to inhibit *E. coli* (4.7 times versus 1.5 times respectively). This is logical in that Ltnα has been shown to have greater solo activity, and can bind to lipid II and prevent peptidoglycan synthesis [7]. However in the presence of polymyxin B/E, Ltnα needs to be added at a 6 times greater concentration to bring about an inhibitory effect equal to that achieved by Ltnα:Ltnβ combined. In contrast, Ltnβ only needs to be added at a 4.7 fold greater concentration to compensate for the absence of Ltnα and thus Ltnβ seems more potent than Ltnα in the presence of either polymyxin. It is not clear if this is due to the potency of Ltnα being slightly compromised by the activity of the polymyxins or is a reflection of a particularly beneficial interaction between these antibiotics and Ltnβ. Additional studies will be required in order to investigate this further.

## Conclusions

Regardless of the mechanism involved, this study documents a means by which lacticin 3147 can be combined with polymyxins in order to effectively inhibit some Gram negative species. There are a number of practical implications to these findings but these will require *in vivo* analysis. One outcome may be to ultimately facilitate the use of lower concentrations of polymyxins in situations where the levels currently employed are of concern from a toxicity perspective. Alternatively, enhancing the spectrum of lacticin 3147 to include Gram negative targets could have benefits with respect to, for example, the treatment of bovine mastitis. While lacticin 3147 has been established as being effective with respect to controlling bovine mastitis caused by Gram positive microorganisms, reducing levels of *S. aureus*, *Streptococcus dysgalactiae* or *Streptococcus uberis*[39,40]*,* mastitis can also be caused by Gram negative species and in particular by *E. coli* species [41,42], against which lacticin 3147 has limited efficacy. *E. coli* can be considered the quintessential environmental pathogen with respect to mastitis. Infections tend to result in acute and often severe clinical mastitis and account for as many as 30% to 40% of clinical mastitis cases [43]. Combining lacticin 3147 with low levels of a polymyxin could provide a means of broadening target specificity, for example in the treatment of mastitis, while keeping the concentrations of antimicrobial employed to a minimum.

## Methods

### Cultures and growth conditions

*Salmonella* Typhimurium UK1, *Salmonella* Typhimurium LT2, *Escherichia coli* 0157:H- , *E. coli* EC101, *E. coli* DH5α (University College Cork (UCC) culture collection) and *Cronobacter sakazakii* 6440 (Dairy Products Research Centre (DPC) collection) were grown in Luria*-*Bertani (LB) broth and agar at 37°C, while *Bacillus cereus* 8079 (DPC collection) and *Enterococcus faecium* strains DO [44], EC538, EC295 and EC725 (British Society for Antimicrobial Chemotherapy (BSAC)) were grown in Brain Heart Infusion (BHI) broth and agar (Oxoid Ltd., Basingstoke, Hampshire, England) at 37°C. *Staphylococcus aureus* strains ST528, ST523, ST530, ST291, ST534 (BSAC) and 5247 (DPC collection) were also grown at 37°C but with aeration in cation supplemented Mueller Hinton broth and Mueller Hinton agar (Oxoid Ltd., Basingstoke, Hampshire, England). *Lactococcus lactis* MG1363 (UCC collection) was grown at 30°C without aeration in M17 broth (Oxoid Ltd., Basingstoke, Hampshire, England) supplemented with 0.5% (wt/vol) glucose (GM17).

### Antimicrobials

Cefoperazone, Cefaclor, Teicoplanin, Bacitricin, Colistin sulphate (polymyxin E), polymyxin B, oxacillin and fusidic acid antimicrobial susceptibility discs were purchased from Oxoid. Polymyxin B and colistin sulphate (polymyxin E) were obtained from Sigma-Aldrich while lacticin 3147 was purified using the following procedure: TYG media (tryptone, 2.5 g/l; yeast extract, 5.0 g/l; glucose, 10 g/l; β-glycerophosphate, 19.0 g/l; MgSO_4_ x 7H_2_O, 0.25g/l; MnSO_4_.4H_2_O, 0.05g/l) was passed through 500g XAD-16 beads (Sigma-Aldrich Company Ltd., Dorset, England) in order to remove all hydrophobic components. An overnight culture of *L. lactis* MG1363.pMRC01.pOM02 [45] was then used to inoculate 1L of the modified TYG broth (1% inoculum) and incubated at 30°C overnight. The cells were subsequently harvested by centrifugation (7000g for 20 min) and resuspended in 250 ml 70% propan-2-ol, pH2 (adjusted to pH2 with addition of conc. HCl). Following stirring at 4°C for four hours the cell debris was removed by centrifugation and the supernatant was subjected to rotary evaporation (50 mbar at 40°C) to reduce the volume to ~60 ml via removal of propan-2-ol. The resultant preparation was applied to a 10 g/60 ml Strata C-18E Giga-Tube (Phenomenex, Cheshire, UK) after pre-equilibration with 60 ml methanol followed by 60 ml water. The column was subsequently washed with 120 ml of 30% ethanol and the lantibiotic was then eluted from the column via addition of 100 ml of 70% propan-2-ol, pH2. From the 100 ml preparation, 20 ml volumes were subjected to rotary evaporation in order to reduce them to ~1.7 ml through removal of propan-2-ol. Aliquots of 1800 μl were then applied to a Phenomenex (Phenomenex, Cheshire, UK) C12 reverse phase (RP)-HPLC column (Jupiter 4 μ 90Å 250 × 10.0 mm, 4 μm) previously equilibrated with 25% propan-2-ol containing 0.1% trifluoroacetic acid (TFA). The column was then developed in a gradient of 30% propan-2-ol containing 0.1% TFA to 60% propan-2-ol containing 0.1% TFA in 4 to 40 min at a flow rate of 1.2 ml/min. Fractions containing Ltnα and Ltnβ were collected after each HPLC run and stored under nitrogen gas. The Ltnα and Ltnβ containing fractions were pooled separately and subsequently subjected to rotary evaporation to remove all propan-2-ol before freeze-drying of the peptides. The Ltnα and Ltnβ peptides were weighed in μg quantities using a Mettler UMT_2_ micro-balance.

### Antibiotic disc-based assessment of antimicrobial sensitivity and synergy

The sensitivities of *S*. Typhimurium LT2, *C. sakazakii* 6440, *S. aureus, and E. faecium* strains to a variety of antibiotics were determined by antibiotic disc diffusion assays as described previously [46]. Briefly, stationary-phase cultures (16 h) were diluted to 10^7^ CFU/ml and swabbed onto Mueller Hinton, LB or BHI agar plates. Six mm antibiotic discs (Oxoid) infused with specific antibiotics were placed on the agar plates. On the same plate lacticin 3147 (1.2, 1.9 or 2.5 μg) was added to a second antibiotic-containing disc and to a blank disc (control). Following overnight incubation (16 h) at 37°C, the resultant zones of inhibition were measured. The antibiotic discs employed included cefotaxime, novobiocin, cefoperazone, teicoplanin, ceftazidime, cefaclor, cephradine, cefaclor (30 μg), bacitracin, imipenem, fusidic acid (10 μg), penicillin G (5 μg), oxacillin (1 μg), colistin sulphate (polymyxin E) (25 μg) and polymyxin B (300U).

### Minimum inhibitory concentrations

MIC determinations were carried out in triplicate in 96 well microtitre plates as previously described by Wiedemann *et al*., 2006. Briefly, bacterial strains were grown overnight in the appropriate conditions and medium, subcultured into fresh broth and allowed to grow to an OD_600nm_ of ~0.5. Serial two-fold dilutions of the lacticin 3147, polymyxin B or colistin sulphate were made in the growth medium of the respective strain. Bacteria were then diluted and added to each microtitre well resulting in a final concentration of 10^5^ cfu/ml in each 0.2 ml MIC test well. After incubation for 16 h at 37°C, the MIC was read as the lowest peptide concentration causing inhibition of visible growth.

### Checkerboard assay for combining antimicrobials

In order to analyse combinations of two different antimicrobials (e.g. X and Y), the minimum inhibitory concentration of each antimicrobial has to be defined against a specific strain. Once this is known a 2-fold serial dilution of X is made horizontally in broth (50 ul) in a microtitre plate beginning at 8 x MIC for X. In a second microtitre plate, a similar dilution of Y is created and then 50 ul of this is added vertically to the original microtitre plate containing the dilution of X. Bacteria were then added in the same fashion as performed for the singular peptide minimum inhibitory assays described previously. Fractional Inhibitory Concentration (FIC) index is defined by the following equation: FIC = FIC_X_ + FIC_Y_ = (X/MIC_X_) + (Y/MIC_Y_). Where (X) is the lowest level of antimicrobial X in combination with another to achieve an inhibitory effect, while (MIC_x_) is the MIC of that antimicrobial alone for the bacterial strain under investigation. FIC index results are interpreted as follows: FIC ≤ 0.5 is synergy, 0.5 < FIC ≤ 0.75 is partial synergy, 0.75 < FIC ≤ 1.0 is additive, FIC >1.0 is indifferent and FIC > 4 is antagonistic [47].

## Authors’ contributions

LD designed experiments, carried out lacticin 3147 purification, antibiotic disc-based, MIC and checkerboard assays and also preparation and drafting of the manuscript. PDC, CH and RPR conceived the study and participated in its design and implementation and reviewed the manuscript. All authors read and approved the final manuscript.
